# Loss of YTHDF1 in gastric tumors restores sensitivity to antitumor immunity by recruiting mature dendritic cells

**DOI:** 10.1136/jitc-2021-003663

**Published:** 2022-02-22

**Authors:** Xiaowu Bai, Chi Chun Wong, Yasi Pan, Huarong Chen, Weixin Liu, Jianning Zhai, Wei Kang, Yu Shi, Masami Yamamoto, Tetsuya Tsukamoto, Sachiyo Nomura, Philip Chiu, Jun Yu, Enders Kwok-wai Ng

**Affiliations:** 1 Institute of Digestive Disease and The Department of Medicine and Therapeutics, State Key Laboratory of Digestive Disease, Li Ka Shing Institute of Health Sciences, CUHK Shenzhen Research Institute, The Chinese University of Hong Kong, Hong Kong, China; 2 Department of Surgery, The Chinese University of Hong Kong, Hong Kong, China; 3 Department of Anatomical and Cellular Pathology, The Chinese University of Hong Kong, Hong Kong, China; 4 Division of Physiological Pathology, School of Veterinary Nursing and Technology, Nippon Veterinary and Life Science University, Tokyo, Japan; 5 Department of Diagnostic Pathology, Fujita Health University School of Medicine, Toyoake, Aichi, Japan; 6 Department of Gastrointestinal Surgery, Graduate School of medicine, The University of Tokyo, Tokyo, Japan

**Keywords:** dendritic cells, immunotherapy

## Abstract

**Background:**

Gastric cancer (GC) is one of the most common cancer worldwide. We analyzed the expression of m^6^A regulatory genes in GC cohorts and revealed that YTHDF1 was uniquely upregulated in GC as compared with adjacent normal tissues. In this study, we analyzed the role of YTHDF1 in GC cells and modulation of the tumor immune microenvironment.

**Methods:**

Three GC cohorts (cohort 1, n=101; cohort 2, n=278, and the Cancer Genome Atlas cohort, n=375) were analyzed for YTHDF1 expression. Function of YTHDF1 in GC was determined in GC cell lines. Role of YTHDF1 in antitumor immunity was investigated in allograft models.

**Results:**

YTHDF1 is upregulated in GC compared with adjacent normal tissues, and high YTHDF1 expression was correlated with poor survival of patients with GC at mRNA (p=0.016) and protein levels (p=0.039). Loss of YTHDF1 in human (AGS, BGC823, MKN74) or mouse (YTN16) GC cell lines inhibited cell growth and colony formation in vitro. Strikingly, syngeneic YTN16 tumors with loss of YTHDF1 underwent complete remission in immunocompetent mice, while a lesser effect was found in immunodeficient mice. Consistently, YTHDF1 loss in GC tumors led to recruitment of mature dendritic cells (DCs) with increased MHCII expression and interleukin-12 (IL-12) secretion, which in turn, promoted CD4^+^ and CD8^+^ T cells infiltration with increased interferon-γ (IFN-γ) secretion. Loss of YTHDF1 mediated the overexpression of IFN-γ receptor 1 and JAK/STAT1 signaling pathway in tumor cells, which might contribute to restored sensitivity to antitumor immunity. In addition, pre-emptive exposure of YTN16 tumors with YTHDF1 loss triggered a potent antitumor immune response on rechallenge with wild-type YTN16 cells, implying that YTHDF1 loss induced a lasting systemic antitumor immunity.

**Conclusions:**

YTHDF1 is overexpressed in GC and promotes GC by inducing cell proliferation and repression of DCs-mediated antitumor immune response. YTHDF1 is a promising therapeutic target for GC treatment.

## Background

Gastric cancer (GC) is the fourth most common cancer worldwide. In northeast Asia, the incidence and mortality rate of GC are the highest in the world. Despite the endeavor in developing novel treatment modalities for GC over the past decades, the clinical outcomes of patients with GC remain poor and mortality-to-incidence ratio continues to be higher than 0.7. Hence, there is a pressing need to investigate the molecular mechanisms underlying gastric carcinogenesis in order to uncover novel therapeutic targets for GC.

M^6^A modification is one of the most important and common messenger RNA modifications, which exerts influential effects on mRNA splicing, stability, and translation. Recent studies have demonstrated that *N*^6^-methyladenosine (m^6^A) modification of mRNA plays an important role in GC.^1–3^ METTL3 is upregulated in GC, and it promotes epithelial-mesenchymal transition (EMT) and metastasis of GC by m^6^A modification of zinc finger MYM-type containing 1 (ZMYM1), leading to ZMYM1-mediated repression of E-cadherin promoter.^1^ YTH domain-containing family protein 1 (YTHDF1), a m^6^A reader, plays a crucial role in gastric carcinogenesis. Recent studies reported that YTHDF1 can promote gastric carcinogenesis via *Wnt* receptor frizzled7 (FZD7) and USP14.^2 3^

Herein, we aimed to determine the role of YTHDF1 in regulating gastric tumor immune microenvironment. We demonstrated that YTHDF1 is overexpressed in GC tissues and it promotes the proliferation of GC cells as an oncogenic factor. In addition to the tumor cell intrinsic effects of YTHDF1, we also revealed that loss of YTHDF1 in GC tumors induced antitumor immunity in syngeneic models, leading to tumor complete remission. Analysis of tumor infiltrating immune cells showed that loss of YTHDF1 promoted the recruitment of mature dendritic cells (DCs), which contributed to increased homing of CD4^+^ and CD8^+^ T-cells to tumors. Loss of YTHDF1 mediated overexpression of interferon (IFN)-γ receptor 1 (IFNGR1) and JAK/STAT1 signaling pathway in tumor cells, which might contribute to restored sensitivity to anti-tumor immunity. These data suggest that targeting of YTHDF1 is a novel strategy for potentiating immunotherapy by restoring sensitivity to anti-tumor immunity in GC.

## Methods

### Patient samples

We collected two cohorts of patients with GC at the Prince of Wales Hospital, Hong Kong: cohort 1 for mRNA expression of YTHDF1, between 2015 and 2018 (n=101) and cohort 2 for protein expression of YTHDF1 measured by IHC staining of tissue microarray, between 1998 and 2006 (n=278). All of the patients were subjected to standard management protocol. All conventional clinical pathological data were evaluated. Human ethics was approved by the Joint Chinese University of Hong Kong—New Territories East Cluster Clinical Research Ethics Committee. We also collected RNA-sequencing and clinical data from patients with GC of the Cancer Genome Atlas (TCGA) database (unpaired group: adjacent normal, n=32, tumor, n=375; paired group: n=27). The correlation of YTHDF1 expression with the infiltration level of DCs was estimated by TIMER2.0. In brief, the immune infiltration level was estimated by R package Immunedeconv. Spearman correlations between the expression of YTHDF1 and the abundance of the immune cell type were then calculated.

### GC cell lines

Human GC cell lines, AGS, BGC823, and MKN74 cells were from the American Type Culture Collection (Manassas, VA); Cell Bank of Chinese Academy of Sciences (Shanghai, China); and Korean Cell Line Bank (Seoul, Korea), respectively. Mouse GC cell line (YTN16) was described previously.^4^

### RNA isolation, cDNA synthesis, and real-time PCR (qPCR)

For tissue samples from our cohort 1, they were individually homogenized with 1 mL of TRIzol reagent (Invitrogen, USA) per 50 mg of tissue. For GC cell lines, we used 1 mL TRIzol reagent per 1×10^6^ cells to lyse the cells. Nanodrop quantification was performed to determine RNA concentration and quality. cDNA was synthesized with PrimeScript RT reagent kit plus gDNA eraser (Takara, Japan). YTHDF1 expression was examined by real-time PCR using QuantStudio 7 Flex PCR System (Thermo Fisher Scientific, Massachusetts, USA). After the reaction is complete, amplification and melting curves were checked and standard curves were plotted.

### Immunohistochemistry (IHC) staining

IHC was conducted on the GC samples from our cohort 2 in tissue microarray (TMA) by Ventana NexES automated Stainer (Ventana). The YTHDF1 primary antibody was diluted at 1:100 (online supplemental table S2). The cytoplasmic expression of YTHDF1 was assessed by assigning a proportion score and an intensity score. The proportion score was according to proportion of tumor cells with positive cytoplasmic staining (0, none; 1, ≤10%; 2, 10–≤25%; 3, >25%–50%; 4, >50%). The intensity score was assigned for the average intensity of positive tumor cells (0, none; 1, weak; 2, intermediate; 3, strong). The cytoplasmic score of YTHDF1 was the product of proportion and intensity scores, ranging from 0 to 12. The cytoplasmic expression was categorized into low expression (score 0–6) and high expression (score 7–12) for survival analysis (online supplemental figure S2).^5^ The sections were evaluated by a pathologist blinded to the nature of the samples.

10.1136/jitc-2021-003663.supp2 Supplementary data online supplemental file 2

10.1136/jitc-2021-003663.supp1 Supplementary data online supplemental file 1

### Primers, Plasmid, shRNAs, and sgRNAs:

qPCR primers,^6^ shRNAs, and sgRNAs^7 8^ are shown in online supplemental table S1. YTHDF1 shRNAs plasmids: pLVshRNA-EGFP-(2A)-Puro and control vector were purchased from Inovogen Technology (Chongqing, China). YTHDF1 sgRNAs and control sgRNA plasmids were synthesized using lenti-CRISPR v2 backbone (Addgene no. 52961).

### Protein extraction, western blot, and cytokine detection

Protein was extracted using CytoBuster Protein Extraction Reagent (Merck, Germany) supplemented with PhosSTOP (Merck, Germany) and Complete Protease Inhibitor Cocktail (Merck, Germany). For western blot, 10–30 µg protein samples were loaded onto 8%–12% SDS-PAGE. Primary antibodies used are listed in online supplemental table S2. Densitometry analysis of western blot bands was performed by Image J (National Institutes of Health, USA). Cytokine levels were determined using Bio-Plex Pro Mouse Cytokine Assay (Bio-Rad, USA).

### Plasmid construction and lenti-virus transduction

Five micrograms of lenti-CRISPR v2 backbone plasmid was digested and dephosphorylated with Esp3I (Thermo Fisher, USA) for 30 min at 37°C. Each pair of oligos were phosphorylated and annealed. The ligation reaction was set up and incubated at room temperature for 10 min and the product was transformed into Stbl3 bacteria (Thermo Fisher, USA). Plasmids of shRNAs and sgRNAs were cotransfected with pMD2.G and psPAX2 plasmids into 293 T cells to generate lentiviral particles, which were used to transduce GC cells. The knockdown or knockout efficiency of YTHDF1 was validated after 3 days of culture by western blot.

### Cell viability assay

Cells (1×10^3^ cells/well) were seeded in 96-well plates. At each time point, 10 µL of 3-(4,5-dimethylthiazol-2-yl)−2,5-diphenyltetrazolium Bromide (MTT) assay (Cat. No. 11465007001, Roche, Switzerland) was added. Absorbance at 570 and 690 nm were then measured with a spectrophotometer. For cell counting, cells (5×10^3^ cells/well) were seeded into 24-well plates, and cell numbers were counted under a light microscopy.

### Colony formation assay

Cells (0.5–1×10^3^ cells/well) were seeded into 6- or 12-well plates. At the end point, cells were washed with PBS and stained with 0.5% crystal violet staining solution. Colonies with more than 50 cells were counted.

### Animal models

MKN74 cells (5×10^6^/tumor) expressing shNC, shYTHDF1-1, or shYTHDF1-2 were suspended in ice-cold 100 µL PBS:Matrigel gel (1:1, v/v) (Corning, USA), and subcutaneously implanted into the right dorsal flank of 4-week-old NOD. Cg-*Prkdc^scid^ Il2rg^tm1Wjl^*/SzJ (NSG) mice. YTN16 cells (5×10^6^/tumor) expressing sgNC, sgYTHDF1-1, or sgYTHDF1-2 in 100 µL PBS:Matrigel gel (1:1, v/v) were subcutaneously injected into the right, left, or both sides of the dorsal flank of 4-week-old NSG mice or C57BL/6 mice to generate syngeneic tumors. At the end point, mice was sacrificed and tumors were processed for flow cytometry analysis, formalin fixation or snap-frozen in liquid nitrogen and storage at −80°C. Tumor volume was measured weekly and tumor weight was recorded at the end point. Tumor volume was calculated as follows: (4π/3)×((Length +Width)/4)^3^. All animal experiments were approved by the Animal Experimentation Ethics Committee of the Chinese University of Hong Kong.

### Flow cytometry

Tissues were dissected into <1 mm^3^ pieces, rinsed in PBS supplemented with 1% collagenase D and 0.005% Dnase I, and placed in a shaker at 225 rpm for 1 hour at 37°C. Cell suspension was filtered with 70 µm strainer, resuspended in 30 mL DMEM with 10% FBS, centrifuged at 2000 rpm for 10 min at 4°C, and then incubated with TruStain FcX PLUS antibody (1:100 dilution) on ice for 10 min. Next, cells were stained with three panels of CD45, CD3ε, CD4, CD8a, Gr-1, CD11b, F4/80, CD11c, MHC-II antibodies (1:100 dilution) in dark for 30 min (online supplemental table S2). Afterwards, the samples were washed two times with 1 mL PBS and centrifuged at 2000 rpm for 10 min. Finally, samples were suspended in 200 µL PBS and detected by BD FACSCelesta flow cytometry. Data were analyzed with the FlowJo Software.

### RNA sequencing

RNA was extracted using TRIzol reagent. RNA sequencing was performed by Novogene (Beijing, China), with 6 GB of data per sample. RNA-seq reads were mapped to human (GRCh38) or mouse reference genomes (GRCm38) by HISAT2 (version 2.1.0). The transcript assembly and quantification were performed by StringTie (v1.3.5). Gene expression was calculated as fragments per kilobase of transcript per million mapped reads) by DESeq2.^9^

### Statistical analysis

Data are presented as mean±SD. Differences between two groups were compared using Student’s t-test. In vitro experiments were performed in triplicates, with at least three independent experiments. Cox regression, Gehan-Breslow-Wilcoxon test, and Kaplan-Meier survival curves were used to analyze the clinical data. Variance between groups were statistically compared. All differences were considered statistically significant if p<0.05. To account for multiple-testing, p values were adjusted using Benjamini-Hochberg false discovery rate correction. GraphPad Prism 8.0 and open-source R software (V.3.5.2) were used to perform statistical analysis.

## Results

### YTHDF1 is overexpressed in GC and associated with poor survival of patients with GC

**Figure 1:**
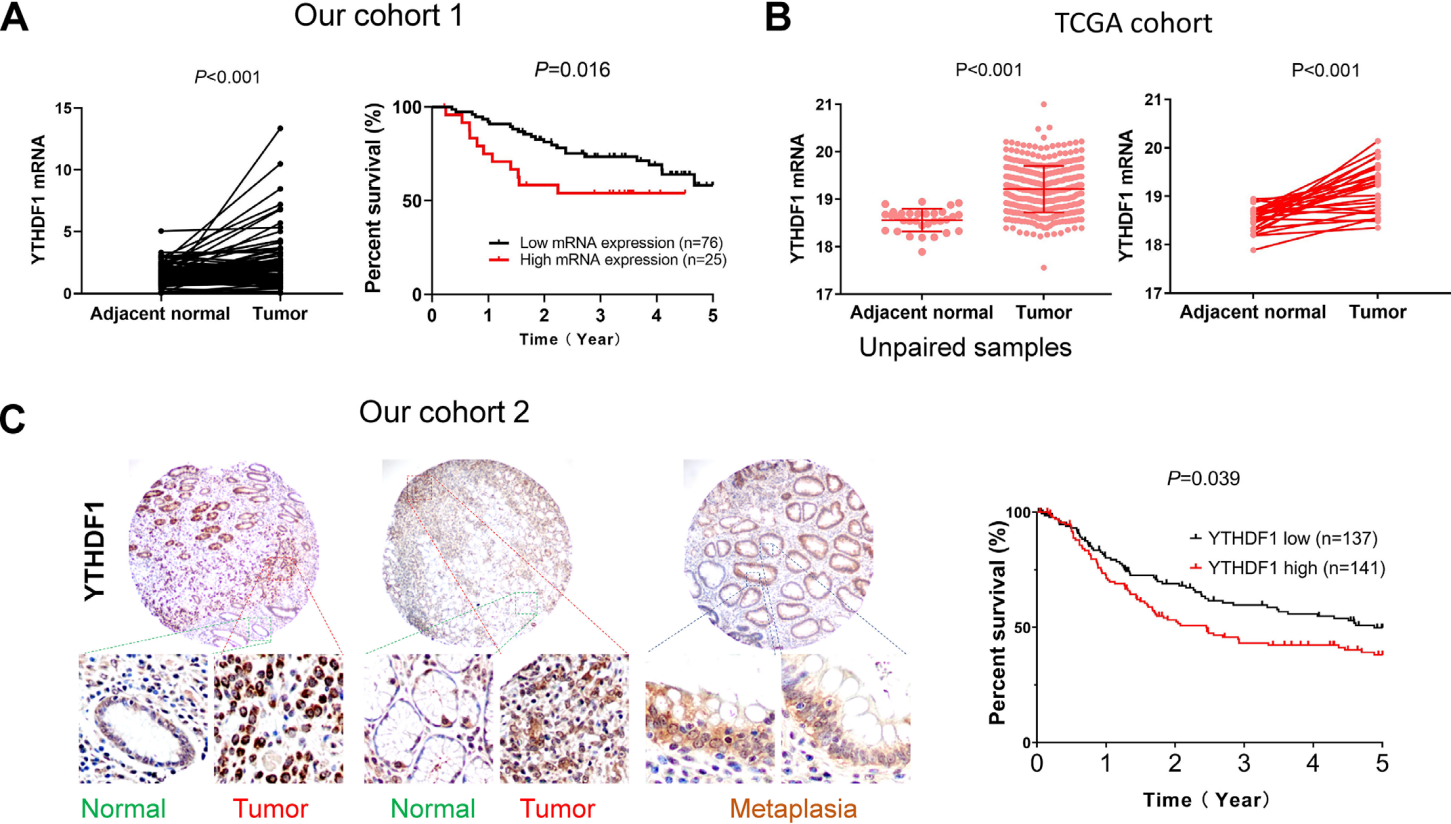
YTHDF1 is overexpressed in GC and its expression is associated with poor survival of patients with GC. (A) Comparison of YTHDF1 mRNA levels between paired GC tumor tissues and adjacent normal tissues from our GC cohort 1 (n=101) and YTHDF1 mRNA expression predicts worse survival using Gehan-Breslow-Wilcoxon test. (B) Comparison of YTHDF1 mRNA levels in TCGA GC cohort (overall cohort: adjacent normal: n=32, tumor: n=375; paired samples: n=27). (C) YTHDF1 protein expression predicts worse survival in patients with GC using Gehan-Breslow-Wilcoxon test. Representative images of TMA in our cohort 2. GC, gastric cancer; TMA, tissue microarray.

We surveyed the expression of m^6^A-related genes in GC tumors compared with adjacent normal tissues in TCGA cohort (online supplemental figure S1), which revealed that YTHDF1 is the most significantly altered m^6^A-related gene in GC and upregulation of YTHDF1 was confirmed both in paired GC cases (n=27, p<001), and overall GC cohort (GC, n=375; adjacent normal, n=32, p<0.001) (figure 1B). Using our cohort 1 of paired GC and adjacent normal tissues (n=101), we validated that YTHDF1 mRNA levels were significantly overexpressed in GC (p<0.001) (figure 1A).

We next sought to determine whether YTHDF1 expression correlates with the survival of patients with GC in our cohorts. Using mean mRNA expression of YTHDF1 in tumor tissues as cut-off, the Kaplan-Meier curves showed that high YTHDF1 mRNA expression was significantly correlated with poor survival for patients with GC in our cohorts 1 (n=101, p=0.0164) (figure 1A). Multivariate Cox regression analysis suggested that high YTHDF1 mRNA expression was an independent poor prognostic factor for patients with GC (p=0.002; OR: 1.274; 95% CI 1.092 to 1.485) (online supplemental table S3). We next evaluated the protein expression by IHC in TMA of our cohort 2 with survival information. We confirmed that YTHDF1 protein expression is associated poor survival of patients with GC (p=0.039) (figure 1, online supplemental figure S2).^5^ These results indicate that YTHDF1 is a potential prognostic factor in GC.

### Loss of YTHDF1 in GC cell lines suppresses cell growth

**Figure 2:**
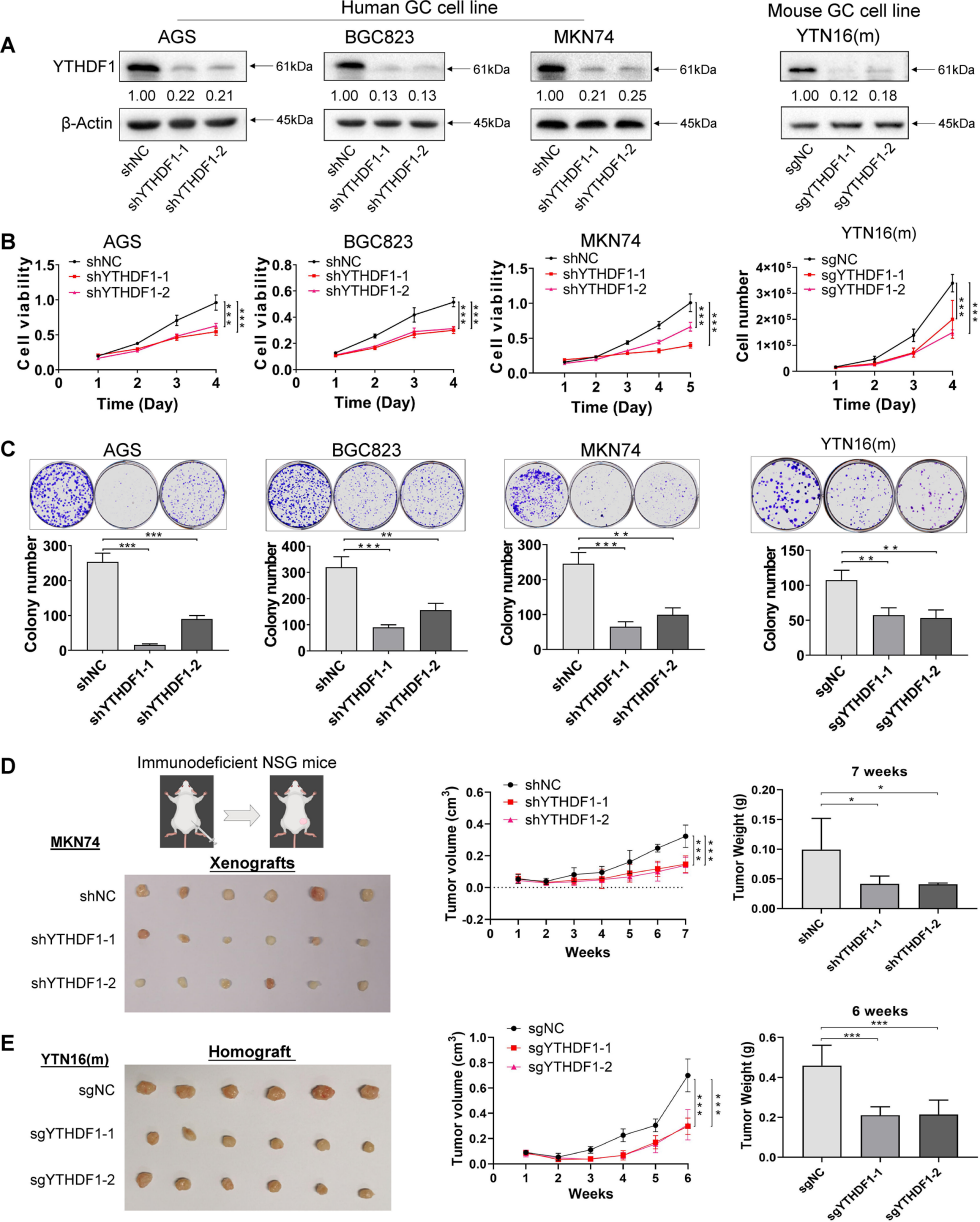
Loss of YTHDF1 in GC cell lines suppresses growth in vitro and tumor growth in immunodeficient mice. (A) Knockdown or knockout of YTHDF1 protein in AGS, BGC823, MKN74, and YTN16 cell lines were confirmed by western blot. (B) Cell viability was determined in GC cell lines with YTHDF1 knockdown or knockout by MTT assay or cell counting. (C) Colony formation assay of GC cells with YTHDF1 knockdown or knockout. (D) MKN74 cells (5×10^6^ cells per tumor) expressing control vector or YTHDF1-shRNA were injected into the right dorsal flanks of NSG mice (n=6 for each group). Mice were sacrificed 7 weeks after injection. Representative tumor images, tumor volume, and tumor weight were shown. (E) YTN16 cells (5×10^6^ cells per tumor) with control vector or knockout of YTHDF1 were injected into the right dorsal flanks of NSG mice (n=6 for each group). Mice were sacrificed 6 weeks after injection. Representative tumor images, tumor volume, and tumor weight were shown. *P<0.05; **p<0.01; ***p<0.001. GC, gastric cancer; NSG, NOD.Cg-*Prkdc^scid^ Il2rg^tm1Wjl^*/SzJ.

High expression of YTHDF1 in tumor tissues implies that YTHDF1 might play an oncogenic role in GC. To determine the biological roles of YTHDF1 in GC, we first examined YTHDF1 protein expression in 12 human GC cell lines (online supplemental figure S3) and showed that YTHDF1 protein expression was relatively high in three GC cell lines AGS, BGC823, MKN74. We therefore selected these cell lines for knockdown studies. Knockdown efficiency of two independent shYTHDF1 was confirmed by western blot (figure 2A). We found that knockdown of YTHDF1 significantly suppressed cell proliferation of human GC cell lines (figure 2B). In a mouse GC cell line YTN16, CRISPR/Cas9-mediated knockout of YTHDF1 (figure 2A) also inhibited cell growth (figure 2B). In support of this, YTHDF1 knockdown or knockout also inhibited colony formation ability in AGS, BGC823, MKN74, and YTN16 cells (figure 2C).

### Loss of YTHDF1 inhibits the growth of GC tumors in immunodeficient mice

To further elucidate the cancer promoting role of YTHDF1 in vivo, we injected MKN74 cells expressing control vector, shYTHDF1-1 or shYTHDF1-2 into the right dorsal flank of NSG mice (figure 2D). MKN74-shYTHDF1-1 and MKN74- shYTHDF1-2 xenografts showed significantly diminished growth compared with controls, both in terms of tumor volume (p<0.001) and weight (p<0.05) (figure 2D). We next sought to validate the effect of YTHDF1 using YTN16 murine GC cells. YTN16 cells with or without YTHDF1 were similarly injected into NSG mice (figure 2E). Consistently, YTHDF1 knockout by two independent sgRNAs significantly inhibited both the size (p<0.001) and weight (p<0.001) of YTN16 tumors in NSG mice (figure 2E). Taken together, these data imply that YTHDF1 functions as an oncogenic factor in GC cells in vivo.

### YTHDF1 modulates adaptive immunity-associated signaling cascades

**Figure 3:**
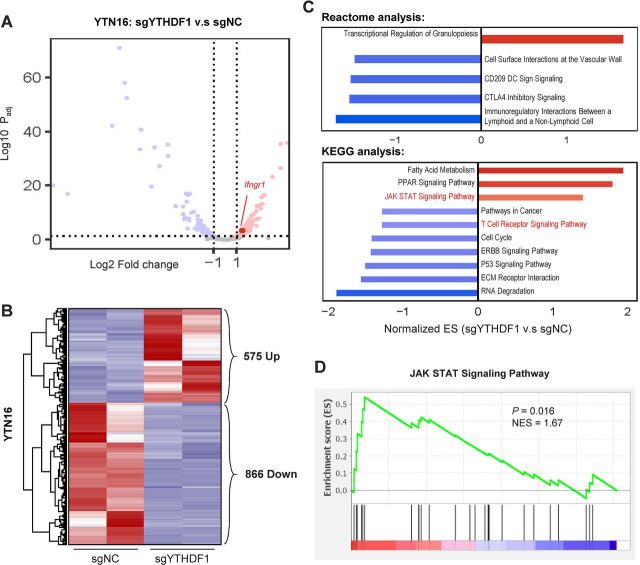
RNA-sequencing of YTN16 cells with loss of YTHDF1 reveals differential regulation of immune-related pathways. (A) Volcano plot and (B) heatmap analysis showing differentially regulated genes in YTHDF1 knockout YTN16 cells. (C) Gene set enrichment analysis of YTN16 (sgYTHDF1 vs sgNC) cells using Reactome and KEGG databases. (D) JAK-STAT signaling pathway was enriched after YTHDF1 knockout in YTN16 cells.

To probe the molecular mechanism underlying YTHDF1 in GC, we performed RNA-sequencing of YTN16 cells expressing sgNC and sgYTHDF1. Volcano plot (figure 3A) and heatmap (figure 3B) analysis revealed 575 and 866 genes that were differentially upregulated and downregulated on YTHDF1 knockout, respectively. We then performed pathway enrichment analysis based on these differentially expressed genes. Gene set enrichment analysis (GSEA) based on Reactome database unraveled immunoregulatory pathways, including CTLA4 checkpoint signaling and CD209 DC signaling as top depleted gene sets after YTHDF1 knockout (figure 3C). Correspondingly, GSEA with the KEGG database identified the enrichment of JAK STAT signaling and T cell receptor signaling that are associated with immune regulation (figure 3C, D), implying that YTHDF1 might exert an immunoregulatory effect in vivo.

### Loss of YTHDF1 eliminates tumorigenesis in immunocompetent mice

**Figure 4:**
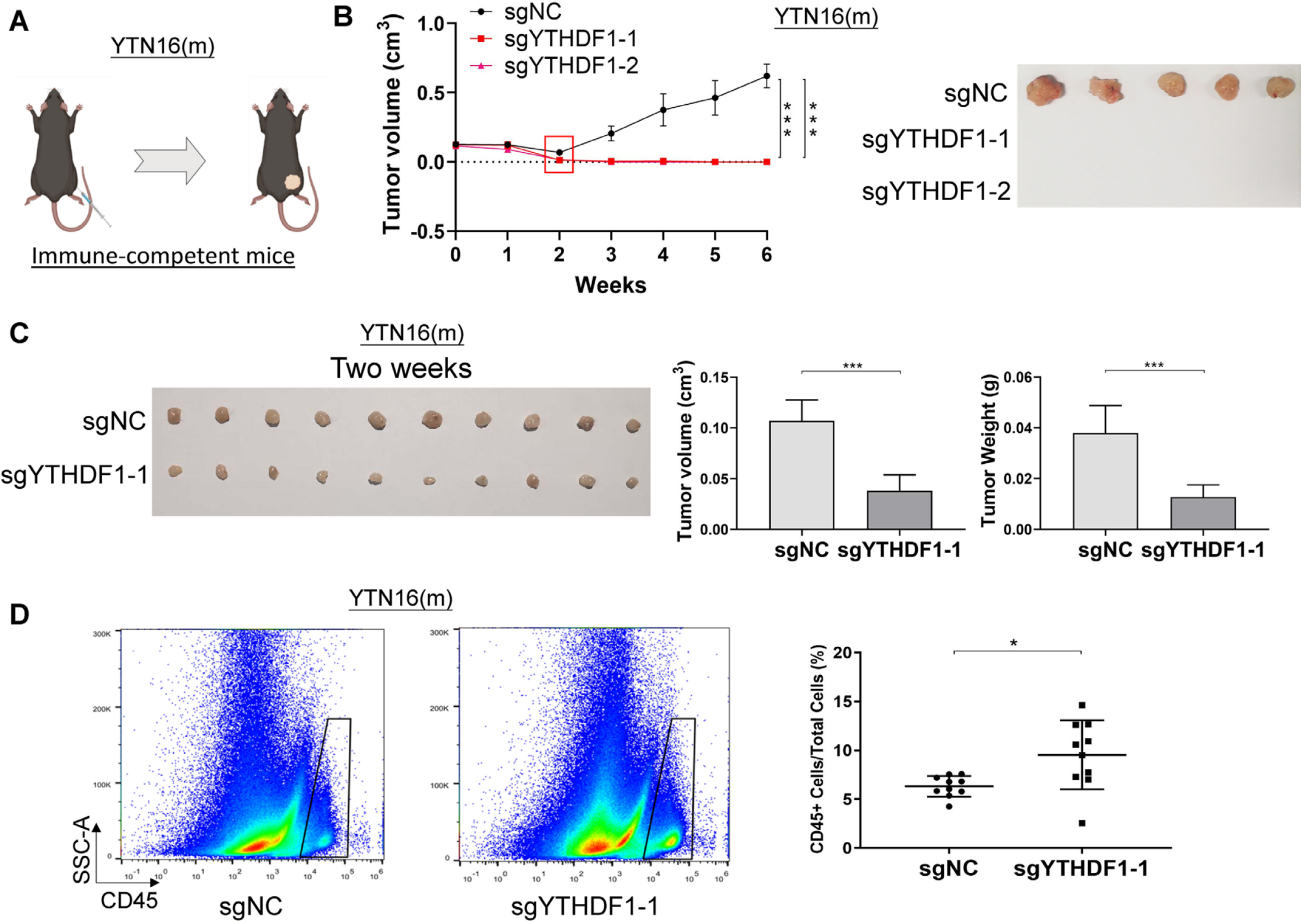
Loss of YTHDF1 induces tumor elimination in syngeneic GC tumors in immunocompetent mice with increased immune cell infiltration. (A) YTN16 cells (5×10^6^ cells per tumor) with control vector or YTHDF1 knockout were injected into the right dorsal flanks of C57BL/6 mice (n=5 for each group). (B) Tumors were measured weekly prior to sacrifice at 6 weeks after injection. Tumor volume and representative tumor images were shown. (C) Control and YTHDF1 knockout YTN16 tumors were harvested at 2 weeks after injection (n=10 for each group). Representative tumor images, tumor volume, and tumor weight were shown. (C) Flow cytometry analysis of infiltrating CD45 positive cells in YTN16 tumors by flow cytometry. The proportion of infiltrating immune cells (CD45^+^ cells) in the tumors was significantly higher in YTHDF1 knockout group than that in the control group. *P<0.05; ***p<0.001.

To ask if the oncogenic effect of YTHDF1 is dependent on a functional immune system, we next used a syngeneic mouse model. We injected YTN16 cells with YTHDF1 knockout (sgYTHDF1-1 and sgYTHDF1-2) or empty control into the dorsal flanks of syngeneic immunocompetent C57BL/6 mice (figure 4A). YTN16 control tumor demonstrated robust growth over the entire study period (figure 4B). On the other hand, YTN16 cells expressing sgYTHDF1-1 or sgYTHDF1-2 formed tumors 1-week postinjection; however, we observed a gradual complete tumor remission in sgYTHDF1 groups beginning at 2-week postinjection, an effect that was maintained until the end point (week 6) (figure 4B). This implies that tumor promoting effect of YTHDF1 is, at least in part, dependent on a functioning immune system.

### Loss of YTHDF1 in tumor cells reactivates adaptive antitumor immunity in vivo

Since YTHDF1 knockout has a considerable greater impact on tumorigenesis in the presence of an intact immune system, we hypothesized that YTHDF1 in tumors might regulate antitumor immunity in vivo. To this end, we implanted YTN16 cells with or without YTHDF1 in immunocompetent C57BL/6 mice. Tumors were harvested 2 weeks after implantation, and the immune cell infiltration in the tumor microenvironment was determined by flow cytometry. Consistently, loss of YTHDF1 caused a significant decrease in both tumor size and tumor weight as compared with control (figure 4C). Flow cytometry revealed that the proportion of the infiltrating immune cells (CD45^+^ cells) in the tumor was significantly higher in YTHDF1 knockout group than that in the control group (p<0.05) (figure 4D).

**Figure 5:**
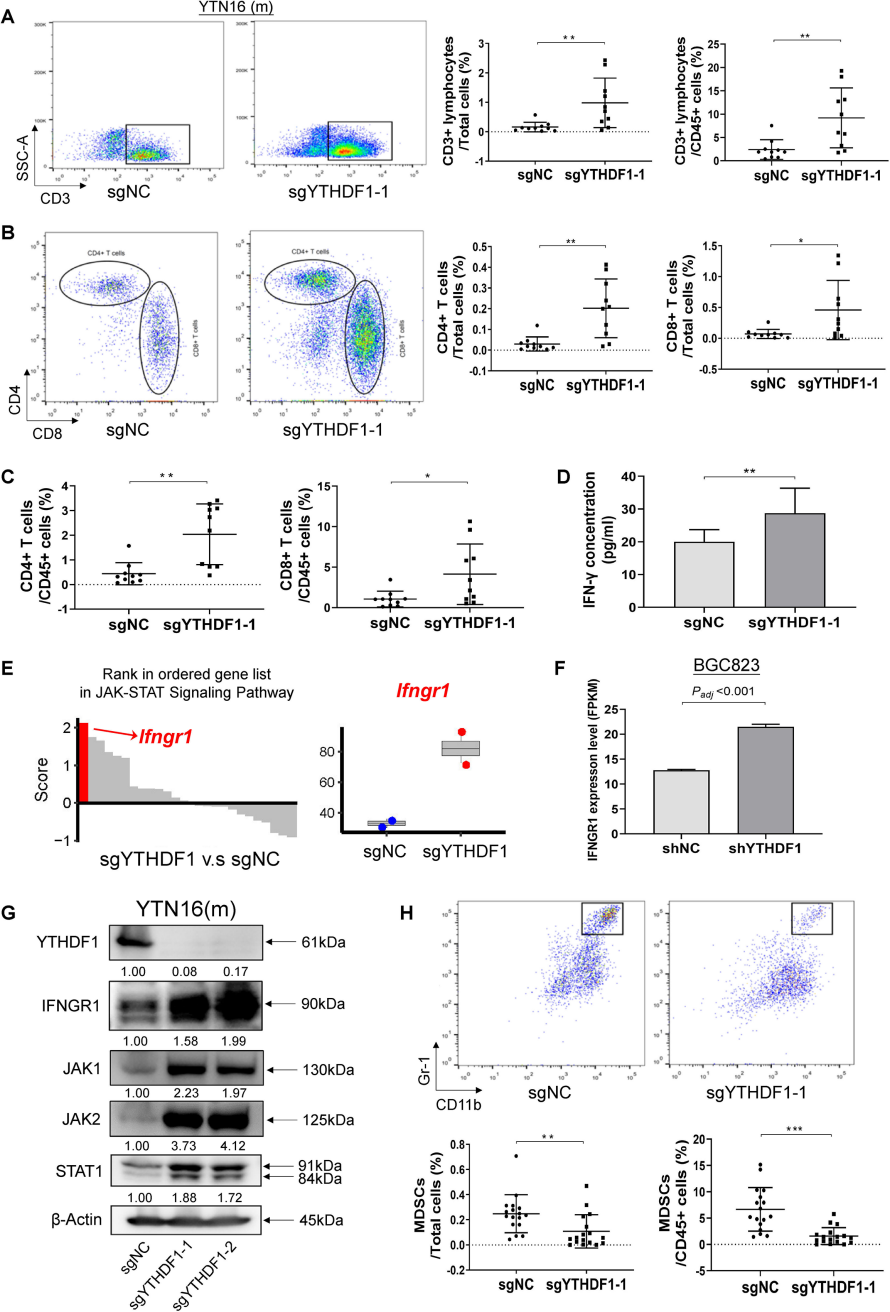
Loss of YTHDF1 in tumor cells activates the adaptive antitumor immunity. (A) Representative images of CD3^+^ lymphocytes infiltration in the YTN16 tumor by flow cytometry, expressed as the percentage of whole tumor or infiltrating immune cell (CD45^+^) population. (B) Representative images of infiltrating CD4^+^ T cells and CD8^+^ T cells by flow cytometry, expressed as the percentage of whole tumor. (C) CD4^+^ T cells and CD8^+^ T cells expressed as the percentage of infiltrating immune cell (CD45^+^) population. (D) Levels of IFNγ in tumors from control and YTHDF1 knockout YTN16 groups. (E) Rank in ordered gene list in JAK-STAT signaling pathway enriched in YTN16 cells with YTHDF1 knockout. The top induced gene in this pathway was Ifngr1. (F) IFNGR1 mRNA expression of BGC823 cell in RNA-sequencing data between two groups. (G) Western blot of IFNGR1 and JAK/STAT1 signaling molecules in GC cells. (H) Representative images of infiltrating MDSCs by flow cytometry, expressed as the percentage of whole tumor or infiltrating immune cell (CD45^+^) population. *P<0.05; **p<0.01, ***p<0.001. IFN, interferon.

T cell (CD3^+^ lymphocyte) is a main component of adaptive antitumor immunity. We thus investigated the infiltration of T lymphocytes in YTN16 tumors. The proportion of CD3^+^ cells in YTN16 tumor and total immune infiltrates (CD45^+^ cells) were both significantly higher in YTHDF1 knockout tumors as compared with control tumors (figure 5A). T lymphocytes mainly consist of T helper cells (CD4^+^) and cytotoxic T cells (CD8^+^). We observed that both the proportion of CD3^+^CD4^+^ T helper cells and CD3^+^CD8^+^ cytotoxic T cells were significantly higher in YTHDF1 knockout tumors as compared with control tumors (figure 5B). Besides, the proportion of T helper cells and cytotoxic T cells in the infiltrating immune cells (CD45^+^ cells) were also significantly higher in YTHDF1 knockout group (figure 5C). IFNγ is secreted predominantly by activated lymphocytes such as CD4^+^ T helper type 1 cells and CD8^+^ cytotoxic T cells.^10–14^ Hence, we measured IFN-γ levels in tumors. Indeed, IFNγ in tumors was significantly higher in YTHDF1 knockout group as compared with control tumors (figure 5D). Interestingly, pathway enrichment analysis showed that Ifngr1 was the top upregulated gene in JAK STAT signaling gene set after YTHDF1 knockout in YTN16 cells (figure 5E). We validated these data in RNA-sequencing data of BGC823 cells (figure 5F). We validated that protein levels of IFNGR1, JAK1, JAK2, and STAT1 were upregulated by YTHDF1 loss in GC cells (figure 5G). Ruxolitinib, a JAK1/2 inhibitor, suppressed STAT1 activation (online supplemental figure S4). Upregulation of IFNGR1 may thus activate JAK-STAT1 of tumor cells, which in turn facilitates effective immune surveillance.

### Loss of YTHDF1 in tumor cells suppresses the infiltration of myeloid-derived suppressor cells

Myeloid-derived suppressor cells (MDSCs) are tumor infiltrating immune cells with immunosuppressive capacity, and accumulating evidence indicates that high infiltration of MDSCs is associated with poor prognosis and therapeutic resistance.^15–18^ We therefore measured MDSCs infiltration in YTN16 tumors by flow cytometry. We observed that proportion of MDSCs in tumor and in infiltrating immune cells were significantly repressed in YTHDF1 knockout tumors as compared with control tumors (figure 5H). This result implies that loss of YTHDF1 in tumor cells promotes adaptive T-cell antitumor immunity and hampers the immunosuppressive cells within the tumor.

### Loss of YTHDF1 in tumor cells induces recruitment of mature dendritic cells *in vivo*

**Figure 6:**
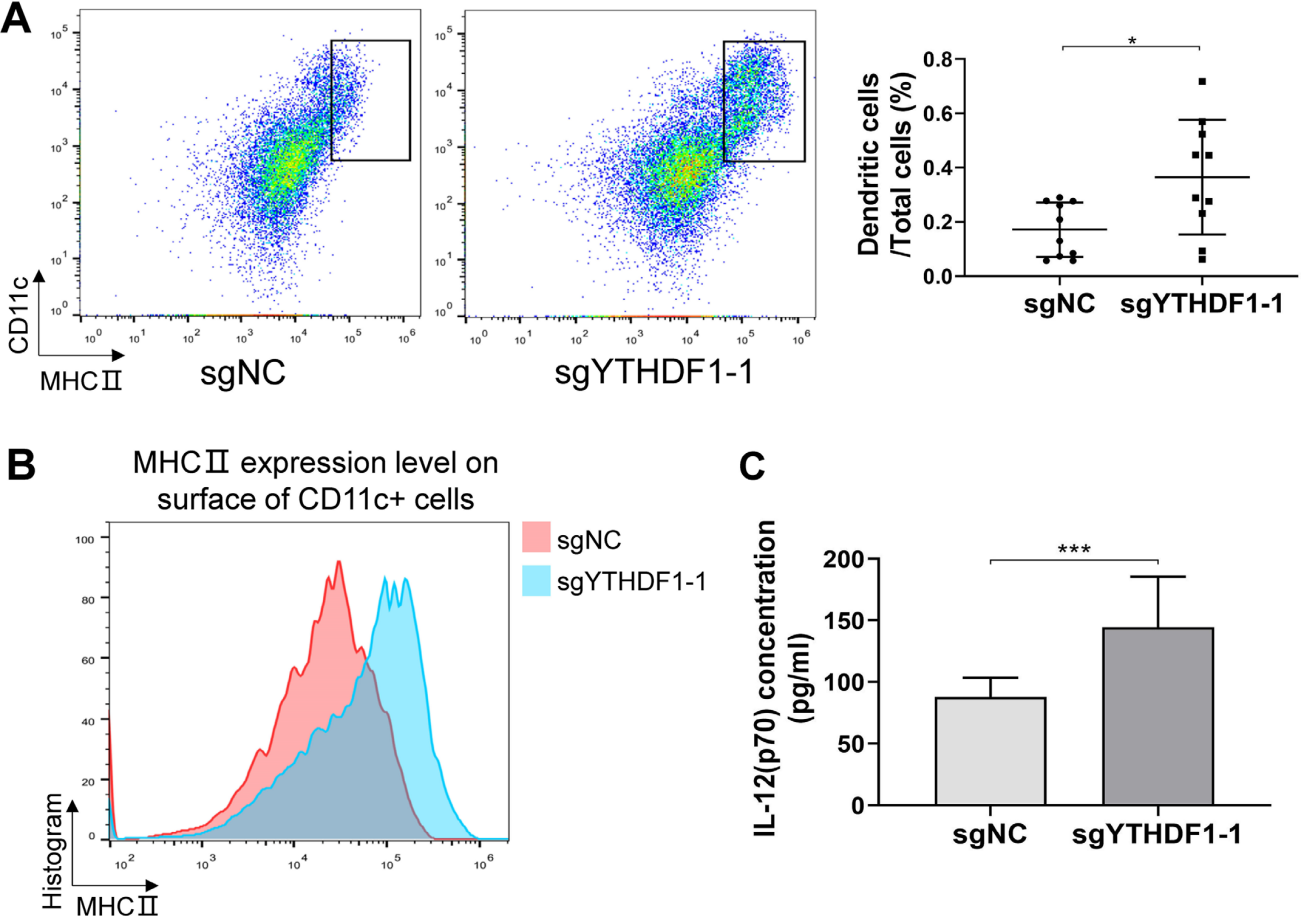
Loss of YTHDF1 in tumor cells recruited more mature DCs in tumors. (A) Representative images of infiltrating DCs as determined by flow cytometry, expressed as the percentage of whole tumor cells. (B) The intensity of surface MHCII molecules in CD11c^+^ cells by flow cytometry. (C) Levels of IL-12(p70) concentration in tumors between two groups. *P<0.05; ***p<0.001. DC, dendritic cell.

The adaptive antitumor immunity is dependent on DCs, which present antigens from tumor cells to prime tumor-specific IFNγ-producing T lymphocytes.^19^ We thus determined DCs in the tumor. We found the proportion of the infiltrating DCs (CD11c^+^MHCII^+^) in tumors was significantly higher in YTHDF1 knockout group than in the control group (figure 6A). Surface MHCII expression of DCs is a fundamental feature of DC maturation.^20^ We analyzed the expression of surface MHCII molecules of infiltrating DCs and found higher surface MHCII expression in DCs from YTHDF1 knockout tumors than control tumors (figure 6B). Mature DCs secrete high levels of interleukin-12 (IL-12(p70)) that enhances adaptive antitumor immunity.^21–23^ In line with increased mature DCs infiltration in YTHDF1 knockout tumors, we demonstrated that IL-12(p70) was elevated in YTHDF1 knockout tumors as compared with control tumors (figure 6C). In addition, DCs tumor infiltration was negatively correlated with the YTHDF1 mRNA expression in TCGA cohort (p<0.0001) (online supplemental figure S5). Collectively, these results suggest that loss of YTHDF1 in tumor cells leads to the recruitment of mature DCs in the tumor, which in turn promote infiltration of T helper cells and cytotoxic T cells, and increased the production of cytotoxic cytokines.

### YTHDF1 expression in tumor cells represses the systemic antitumor immune response

**Figure 7:**
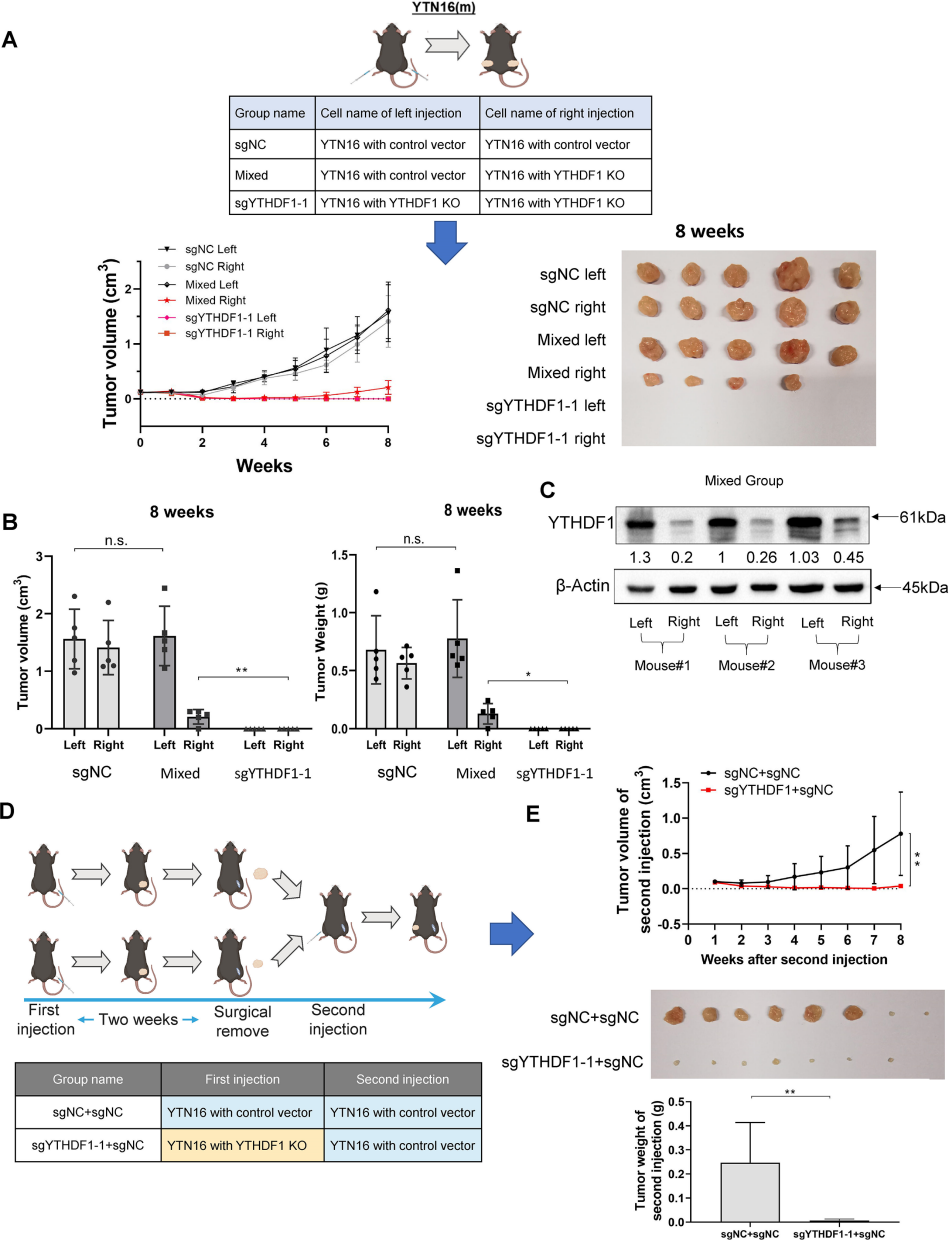
YTHDF1 expression in GC tumor cells exert systemic effects on host antitumor immunity. (A) YTN16 cells (5×10^6^ cells per tumor) with control vector or YTHDF1 knockout were injected into dorsal flanks of C57BL/6 mice (n=5 for each group) as indicated. Tumors were measured weekly prior to sacrifice at 8 weeks after cell injection. Tumor images were shown. (B) Tumor volume and tumor weight measurements suggested that the growth of YTHDF1 knockout tumors in the mixed group was significantly increased compared with that in sgYTHDF1-1 only group (right tumor in mixed group *vs* right tumor in sgYTHDF1-1 group). (C) Protein expression of YTHDF1 of the left-side and right-side tumors in the mixed group. (D) Mice were divided into two groups and injected the YTN16 cells with or without YTHDF1 into the right dorsal flanks of mice (n=8 for each group). After 2 weeks, tumors were surgically completely removed and YTN16 cells with control vector were injected into left dorsal flanks of both groups of mice. (E) Tumor growth on the left dorsal flank was monitored weekly. YTN16 tumor growth with control vector on the left dorsal flank was impaired in mice previously injected with YTN16 knockout tumors (sgYTHDF1−1+sgNC) as compared with those injected with control tumors (sgNC +sgNC). *P<0.05; **p<0.01.

To evaluate whether YTHDF1 expression in tumors affects antitumor immune response in a systemic fashion, we injected YTN16 cells expressing sgNC or sgYTHDF1 in the same mice. Mice were divided into three groups: sgNC, mixed, and sgYTHDF1-1 (figure 7A). In sgNC and sgYTHDF1-1 groups, either cells were injected into both flanks of C57BL/6 mice. In the mixed group, sgNC and sgYTHDF1-1 cells were injected into the left and right flanks of C57BL/6 mice, respectively. In agreement with that in figure 4, tumor growth was observed in sgNC group, but not in sgYTHDF1-1 group (figure 7A, B). Interestingly, in mixed group, significant tumor formation was found even for sgYTHDF1-1 expressing cells (figure 7A, B). Western blot confirmed that sgYTHDF1-1 tumor retained the loss of YTHDF1 protein (figure 7C). This result implies that sgNC tumors may cause a systemic repression in antitumor immunity, which in turn is permissive for the survival of sgYTHDF1-1 tumors.

### Loss of YTHDF1 in tumor cells mediate durable antitumor immune response that rejects subsequent tumor engraftment in vivo

Given that the loss of YTHDF1 eliminated gastric tumors in immunocompetent hosts, we next wondered if it can trigger a durable, systemic antitumor immune response that prevents future tumor implantation.^24 25^ In a nutshell, we divided the mice into two groups and injected the YTN16 cells with or without YTHDF1 into the right dorsal flanks of mice, respectively. Then, tumor nodules formed at the right dorsal flanks were surgically completely removed at 2-week postinjection. This was followed by injection of YTN16-sgNC cells into the left flanks (figure 7D). Tumor growth on the left dorsal flank was then monitored. As shown in figure 7E, prior injection of YTN16 cells with sgYTHDF1-1 was able to confer the host antitumor ability to reject the subsequent implantation of tumor cells with normal YTHDF1 expression. These findings indicated that YTN16 cells with loss of YTHDF1 could trigger systemic antitumor immune responses of the hosts sufficiently to reject further tumor engraftment even with tumor cells of high YTHDF1 expression.

## Discussion

In this study, we identified m^6^A mRNA reader YTHDF1 as the top deregulated m^6^A-related gene and a prognostic factor that predicts poor survival of GC. In vitro and in vivo experiments demonstrated the oncogenic role of YTHDF1 in GC. Apart from its tumor cell intrinsic role, we demonstrated that YTHDF1 expression in GC can suppress antitumor immunity by preventing the recruitment of mature DCs in tumors, thereby suppressing adaptive immunity. Consequently, the loss of YTHDF1 in tumor cells triggered collateral attack to gastric tumors, leading to tumor elimination. Our work underlies the YTHDF1 as a potential therapeutic target in GC.

Aberrant regulation of m^6^A modification of mRNA is increasingly recognized to be a pivotal event in multiple cancers. We systematically screened key m^6^A regulators in GC, revealing that YTHDF1 is the top candidate overexpressed in GC compared with adjacent normal tissues. Moreover, YTHDF1 overexpression predicts poor survival of patients with GC, inferring that YTHDF1 may exert protumorigenic role in GC. Consistent with this hypothesis, YTHDF1 knockdown or knockout inhibited GC cell growth and colony formation in vitro, and induced modest growth inhibition of subcutaneous xenograft models in immunodeficient mice. This suggests that YTHDF1 is important in GC initiation by promoting GC cell growth. Collaborating our observations, recent reports have demonstrated tumor intrinsic effects of YTHDF1 in gastric tumorigenesis by modulating the translation of downstream oncogenic factors, including FZD7,^2^ USP14,^3^ and SPHK2.^26^ Taken together, YTHDF1 functions as an oncogene in GC.

To our surprise, RNA-seq of YTHDF1 knockout GC cells revealed that the top depleted pathways are associated with immune regulatory pathways and DCs. Consistent with this observation, profiling of tumor immune microenvironment revealed that loss of YTHDF1 in GC cells induced adaptive antitumor immunity, characterized by the increased mature DCs, CD4^+^, and CD8^+^ T lymphocytes, concomitant with lower immunosuppressive MDSCs. Concordantly, we found that loss of YTHDF1 in murine GC cells induced complete tumor remission in immunocompetent mice, but only moderately inhibited tumor growth in NSG mice, further suggesting that modulation of the host immunity plays a key role in YTHDF1 mediated gastric tumor progression in vivo.

To mount an adaptive immune response, DCs must incorporate antigens from tumor cells, acquire the competence of antigen processing in a maturation step and present antigenic peptides bind to major histocompatibility complex (MHC) molecules to recognition by specific T cells.^27^ Loss of YTHDF1 in GC tumor cells increased tumor infiltrating mature DCs and boosted the expression of surface MHCII molecules in intratumoral DCs, thereby promoting an effective adaptive immune response. In line with activation of DCs-dependent adaptive immunity, loss of YTHDF1 induced activation of CD4^+^ and CD8^+^ T cells, as evidenced by their increased infiltration and secretion of key cytotoxic cytokines, such as IFNγ.^28 29^ Reports by others also indicate that intratumoral mature DCs as a barometer for effective antitumor immunity. Indeed, elevated mature DCs is associated with favorable prognosis in multiple malignancies, including ovarian cancer,^29^ colorectal cancer^30^ and non-small-cell lung cancer.^31^ These data collectively suggest that YTHFD1 also promotes GC at later stages by suppressing the adaptive immune response.

With regard to the mechanism that caused the infiltration of mature DCs, we demonstrated that loss of YTHDF1 in tumor cells could increase the expression of IFNGR1 in tumor cells. Previous studies showed that IFNγ receptor deficient (IFNGR1^-/-^) mice develop more tumors than wild-type mice and that the IFNγ- responsiveness of the tumor cell is essential for effective immune recognition.^32 33^ Hence, upregulated IFNGR1 in tumor cells can potentially collaborate with lymphocytes to shape the immunogenic phenotype of tumors and facilitate the effective immune recognition.^24 32 33^

Recently, cancer immunotherapy has achieved unprecedented success in the clinic. Most of the current strategies focus on the reactivation antitumor T cell immunity via checkpoint blockade;^25 34^ however, response rates remain limited and alternative strategies is urgently needed. Among them, DC vaccines may elicit and improve antitumor T cell immunity.^35 36^ Wculek *et al* found that dead tumor antigen activates DCs ex vivo, and on adoptive transfer in vivo, these DCs induce strong CD8^+^ T cell responses through antigen cross-presentation and halt the progression of engrafted cancer models. Herein, we revealed that prior inoculation of YTN16 cells with loss of YTHDF1 was able to trigger the host’s antitumor immunity to reject subsequent injection with cells that express normal YTHDF1 levels. These collectively suggest that YTHDF1 manipulation might modulate tumor immunogenicity via its effect on DCs. Thus far, clinical responses of DCs-based immunotherapy have been disappointing due to inefficient DCs activation.^37^ Targeting of YTHDF1 in tumor cells may thus synergize DCs immunotherapy. For example, ex vivo DCs vaccination with GC cells with YTHDF1 knockout could be a promising choice to improve DCs antigen loading and activation, resulting in more effective DC vaccines. Another potential strategy is to directly knockdown YTHDF1 in gastric tumors by in vivo siRNA to promote DCs infiltration and activation, thereby promoting antitumor immune response.

The impact of our findings was further strengthened by the clinical significance of YTHDF1. YTHDF1 is consistently overexpressed in GC as compared with their adjacent normal tissues in independent GC patient cohorts. Moreover, YTHDF1 mRNA and protein expression were both associated with the poor survival of patients with GC. Hence, YTHDF1 might be a useful prognostic biomarker for the differentiation of patients according to their survival outcomes.

In summary, YTHDF1 is overexpressed in GC and exerts a protumorigenic role. Loss of YTHDF1 restores sensitivity to antitumor immunity via the recruitment of DCs, which in turn activates CD4^+^ and CD8^+^ T cells, culminating in tumor remission. In addition, loss of YTHDF1 mediated the overexpression of IFNGR1 and JAK1/2-STAT1 pathway in tumor cells, which might contribute to restored sensitivity to antitumor immune response (online supplemental figure S6). Our results suggest a novel strategy for effective immunotherapy in GC through targeting of YTHDF1 to boost adaptive antitumor immunity.

## Data Availability

Data are available on reasonable request. All data will be available on request from the corresponding author.
