# Supplementary material for: Loss of YTHDF1 in gastric tumors restores sensitivity to antitumor immunity by recruiting mature dendritic cells
Source: J Immunother Cancer. 2022 Feb 22;10(2):e003663. doi: 10.1136/jitc-2021-003663 (PMC9066370; doi:10.1136/jitc-2021-003663)
Supplement: Supplementary data [file jitc-2021-003663supp002.pdf]

Supplementary Table 1. Primers, shRNAs, and sgRNAs used in this study

Name	Sequence
YTHDF1(human) forward	5'-CAAGCACACAACCTCCATCTTCG-3'
Reverse	5'-GTAAGAAACTGGTTCGCCCTCAT-3'
GAPDH (human) forward	5'- AGAAGGCTGGGGCTCATTTG-3'
Reverse	5'-GAGGGGCCATCCACAGTCTTC-3'
shYTHDF1-1 target sequence	GGATACAGTTCATGACAATGA
shYTHDF1-2 target sequence	GGCGTGTGTTCATCATCAAGA
shNC target sequence	GTTCTCCGAACGTGTCACG
sgYTHDF1-1 sequence	CACCGAAGCATGTCGGCCACCAGCG
sgYTHDF1-2 sequence	CACCGTAGGTAGTGAGATACGGGAT
sgNC sequence	CACCGCGAGGTATTCGGCTCCGCG

Supplementary Table 2. Antibodies used in this study

Antibodies	Supplier	Reference
YTHDF1	Proteintech	17479-1-AP
YTHDF1	Abcam	ab220162
IFNGR1	Proteintech	10808-1-AP
JAK1	Cell Signaling	3344
JAK2	Cell Signaling	3230
STAT1	Cell Signaling	14994
β-Actin	Cell Signaling	4970
TruStain FcX™ PLUS (anti-mouse CD16/32)	Biolegend	155603
CD45	Biolegend	103138
CD3ε	Biolegend	100308
CD4	Biolegend	100422
CD8a	Biolegend	100706
Gr-1	Biolegend	108408
CD11b	Biolegend	101206
F4/80	Biolegend	123114
CD11c	Biolegend	117307
MHCⅡ	Biolegend	107631

Supplementary Table 3. Univariate and multivariate Cox regression analysis of YTHDF1 mRNA and clinicopathological parameters in predicting the survival of patients with gastric cancer in our cohort 1

Variable	Univariate COX regression		Multivariate COX regression	
	OR (95% CI)	*P*-value	OR (95% CI)	*P*-value
**Age**	1.025(0.995-1.055)	0.106	1.026(0.991-1.062)	0.149
**Gender**				
Female				
Male	0.613(0.316-1.191)	0.149	0.471(0.214-1.036)	0.061
***H.pylori* infection**				
no				
yes	0.879(0.396-1.951)	0.751	0.645(0.248-1.675)	0.368
**Anatomic subdivision**				
Cardia/proximal				
Fundus/body	0.648(0.271-1.549)	0.330	0.868(0.275-2.741)	0.810
Antrum/distal	0.681(0.281-1.649)	0.394	1.045(0.335-3.259)	0.939
**Histological grade**				
Moderately differentiated				
Poorly differentiated	1.377(0.516-3.672)	0.523	1.540(0.528-4.488)	0.429
**Lauren classification**				
Intestinal type				
Mixed type	0.805(0.287-2.263)	0.681	1.216(0.370-3.998)	0.748
Diffuse type	0.993(0.466-2.116)	0.986	1.064(0.421-2.691)	0.895
**Pathology primary tumor**				
T1				
T2	1.472(0.201-10.773)	0.703	0.871(0.084-9.087)	0.908
T3	5.988(1.318-27.214)	0.020	4.602(0.223-95.043)	0.323
T4	12.418(2.766-55.752)	0.001	5.752(0.251-131.842)	0.274
**Spread to lymph nodes**				
N0				
N1	2.785(0.660-11.758)	0.163	1.620(0.330-7.965)	0.552
N2	7.937(2.143-29.395)	0.002	9.030(0.509-160.254)	0.134
N3	10.558(3.072-36.285)	<0.001	9.897(0.526-186.076)	0.126
**Pathologic stage**				
**I**				
**II**	9.072(1.134-72.558)	0.038	8,185(1.019-65.761)	0.048
**III**	26.278(3.483-198.235)	0.002	23.129(3.041-175.891)	0.002
**YTHDF1 mRNA expression**	1.344(1.155-1.565)	<0.001	1.274(1.092-1.485)	0.002
