# Supplementary material for: Loss of YTHDF1 in gastric tumors restores sensitivity to antitumor immunity by recruiting mature dendritic cells
Source: J Immunother Cancer. 2022 Feb 22;10(2):e003663. doi: 10.1136/jitc-2021-003663 (PMC9066370; doi:10.1136/jitc-2021-003663)
Supplement: Supplementary data [file jitc-2021-003663supp001.pdf]

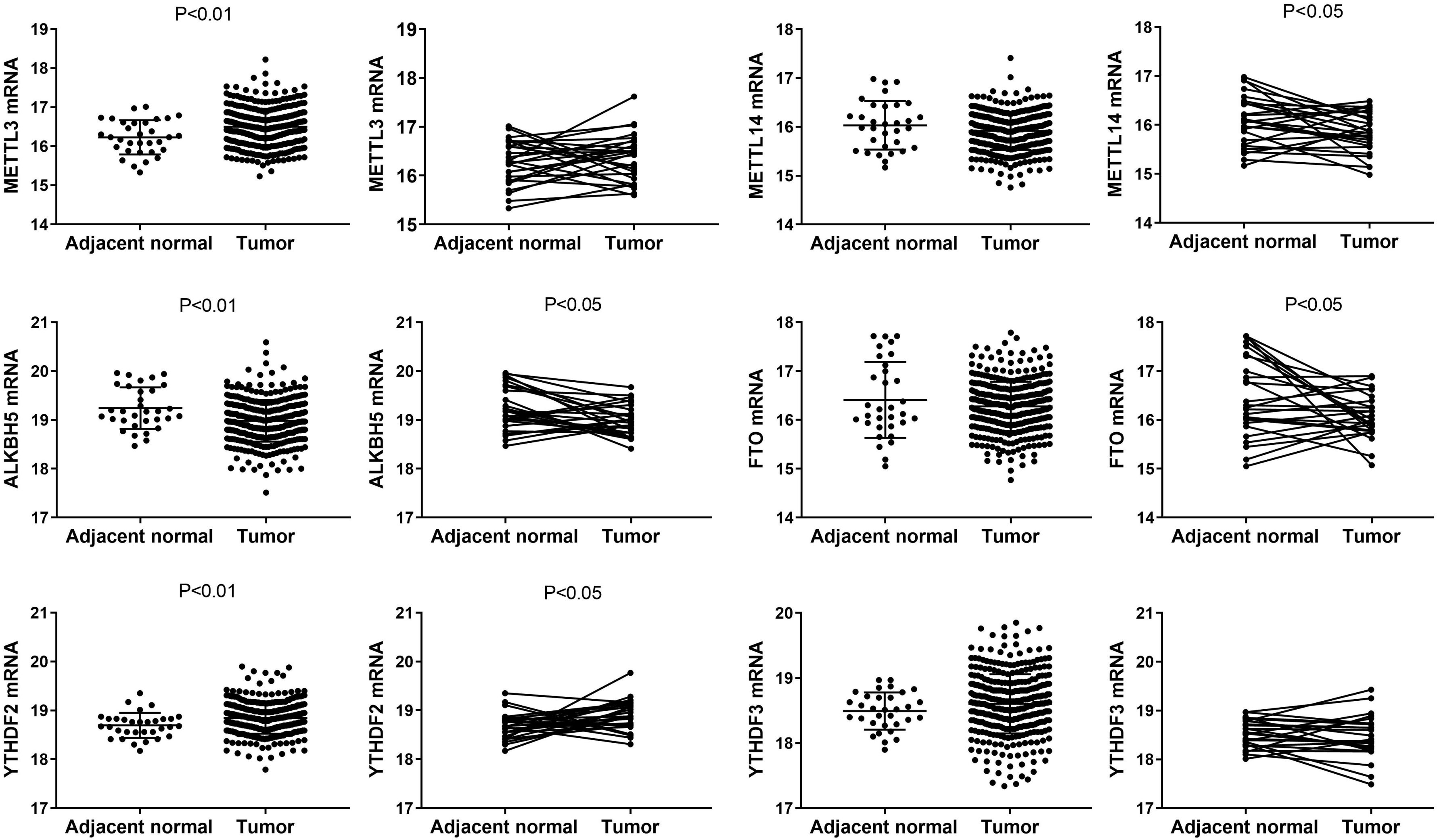

Supplementary figure 1. mRNA expression of m^{6}A-regulated genes mRNA in GC tumor tissues and adjacent normal tissues using TCGA database. Unpaired group (Adjacent normal, n=32, Tumor, n=375); paired group (n=27).

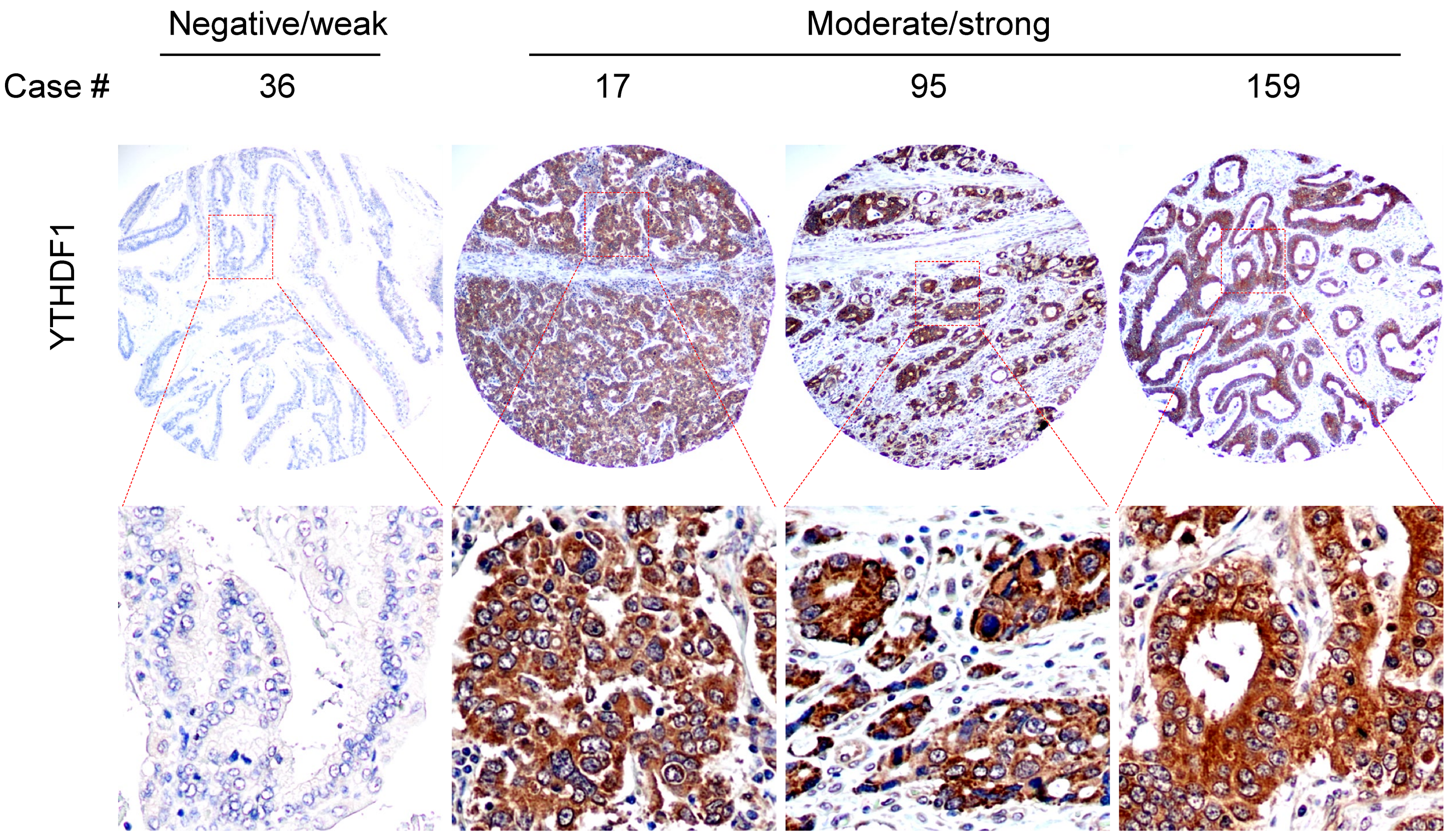

Supplementary figure 2. Representative images of IHC for YTHDF1. Score method: negative/weak expression = low expression; moderate/strong expression = high expression. We define “0-6” as low expression and “7-12” as high expression.

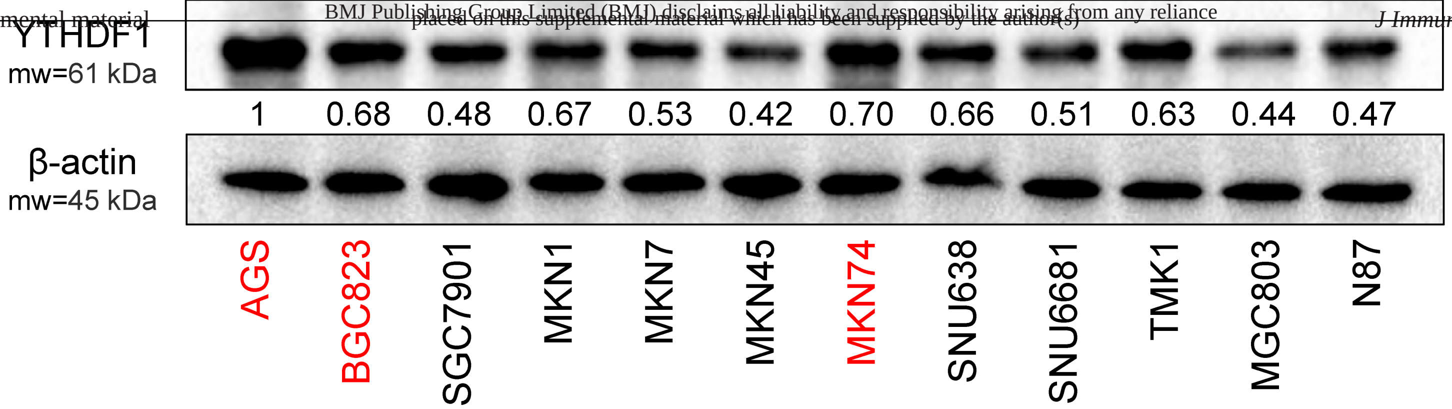

Supplementary figure 3. Protein expression of YTHDF1 in 12 human gastric cancer cell lines.

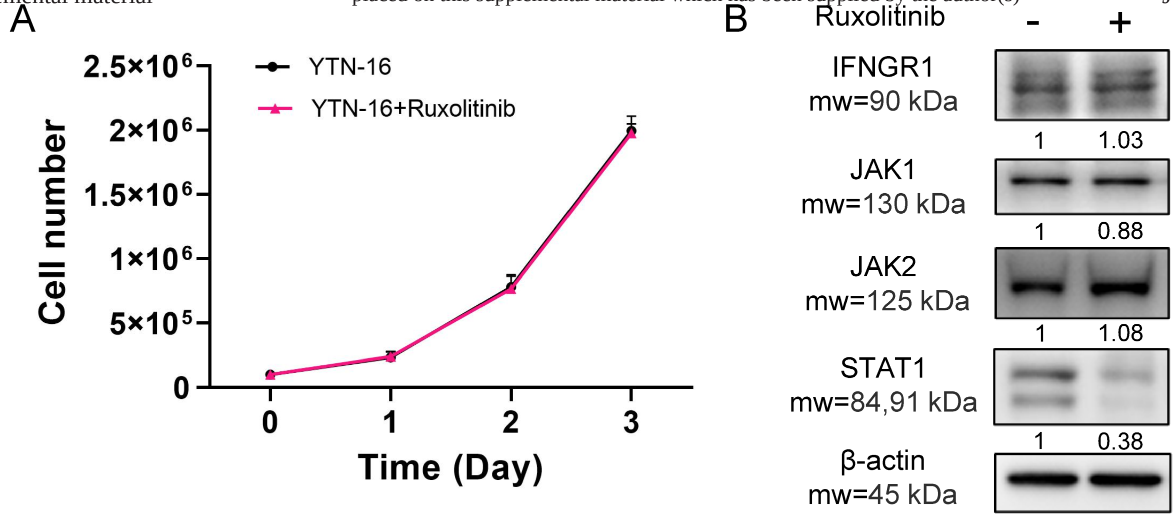

Supplementary figure 4. Effect of Ruxolitinib on GC cell. (A) Cell viability was determined in YTN16 cell (1×10^5 cells) with or without Ruxolitinib (5μM) by cell counting. **(B)** Western blot of IFNGR1 and JAK/STAT1 signaling molecules in YTN16 cell treated with or without Ruxolitinib (5μM) for 24h.

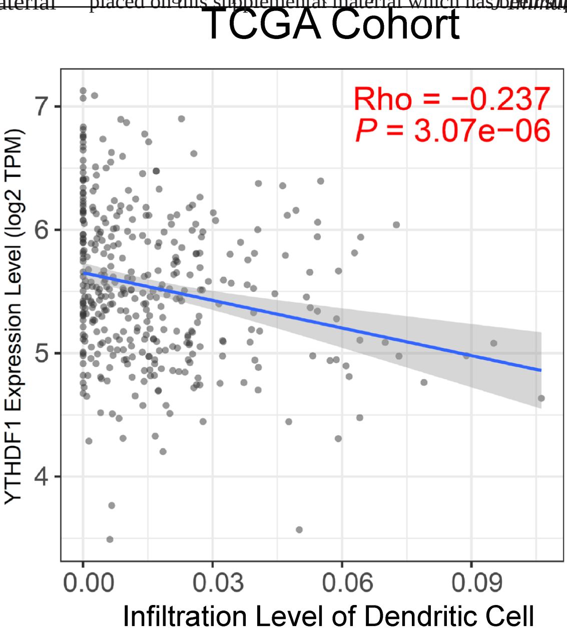

Supplementary figure 5. Correlation analysis of YTHDF1 mRNA expression and dendritic cell infiltration in TCGA database.

BMJ Publishing Group Limited (BMJ) disclaims all liability and responsibility arising from any reliance placed on this supplemental material which has been supplied by the author(s)

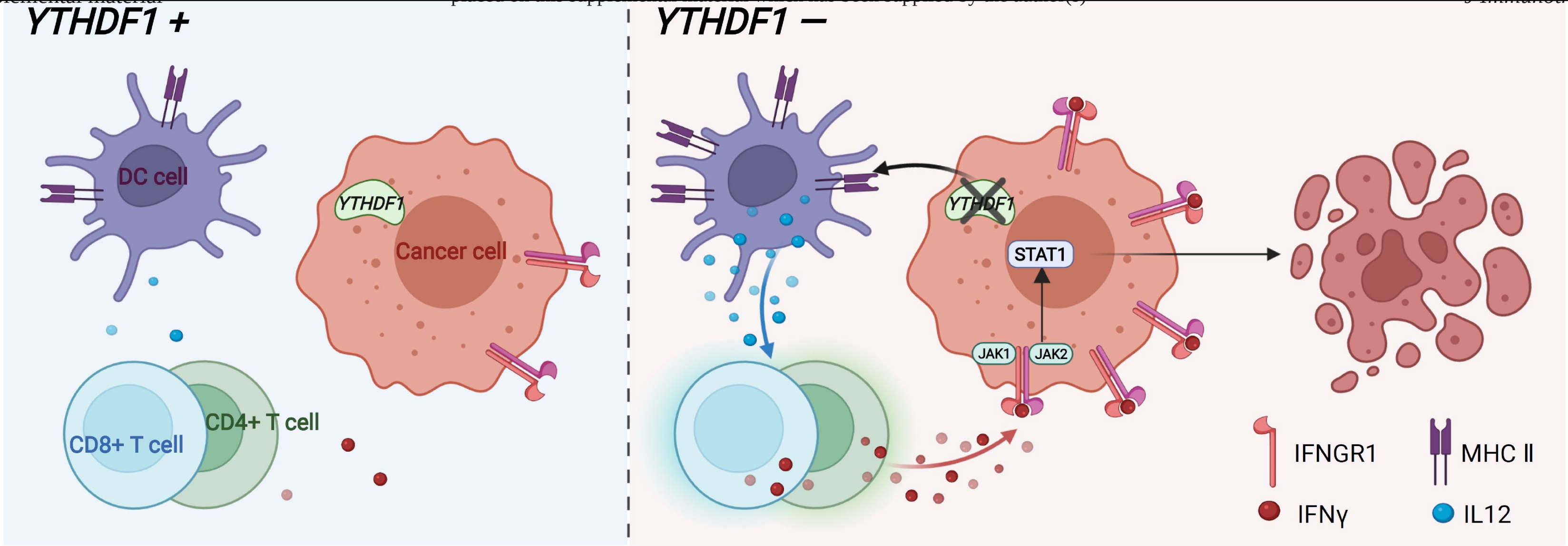

Supplementary figure 6. Overview of the graphic abstract. YTHDF1 loss in gastric cancer tumors led to recruitment of mature dendritic cells (DCs) with increased MHCII expression and interleukin-12 (IL-12) secretion, which in turn, promoted $CD4^{+}$ and $CD8^{+}$ T cells infiltration with increased interferon-γ (IFN-γ) secretion. In addition, loss of YTHDF1 mediated the overexpression of IFN-γ receptor 1 (IFNGR1) and JAK1/2-STAT1 pathway in tumor cells, which might contribute to restored sensitivity to anti-tumor immunity. Created with BioRender.com
